# Supplementary material for: Neurexin-3 subsynaptic densities are spatially distinct from Neurexin-1 and essential for excitatory synapse nanoscale organization in the hippocampus
Source: Nat Commun. 2023 Aug 5;14:4706. doi: 10.1038/s41467-023-40419-2 (PMC10404257; doi:10.1038/s41467-023-40419-2)
Supplement: Supplementary file 1 — Supplementary Information [file 41467_2023_40419_MOESM1_ESM.pdf]

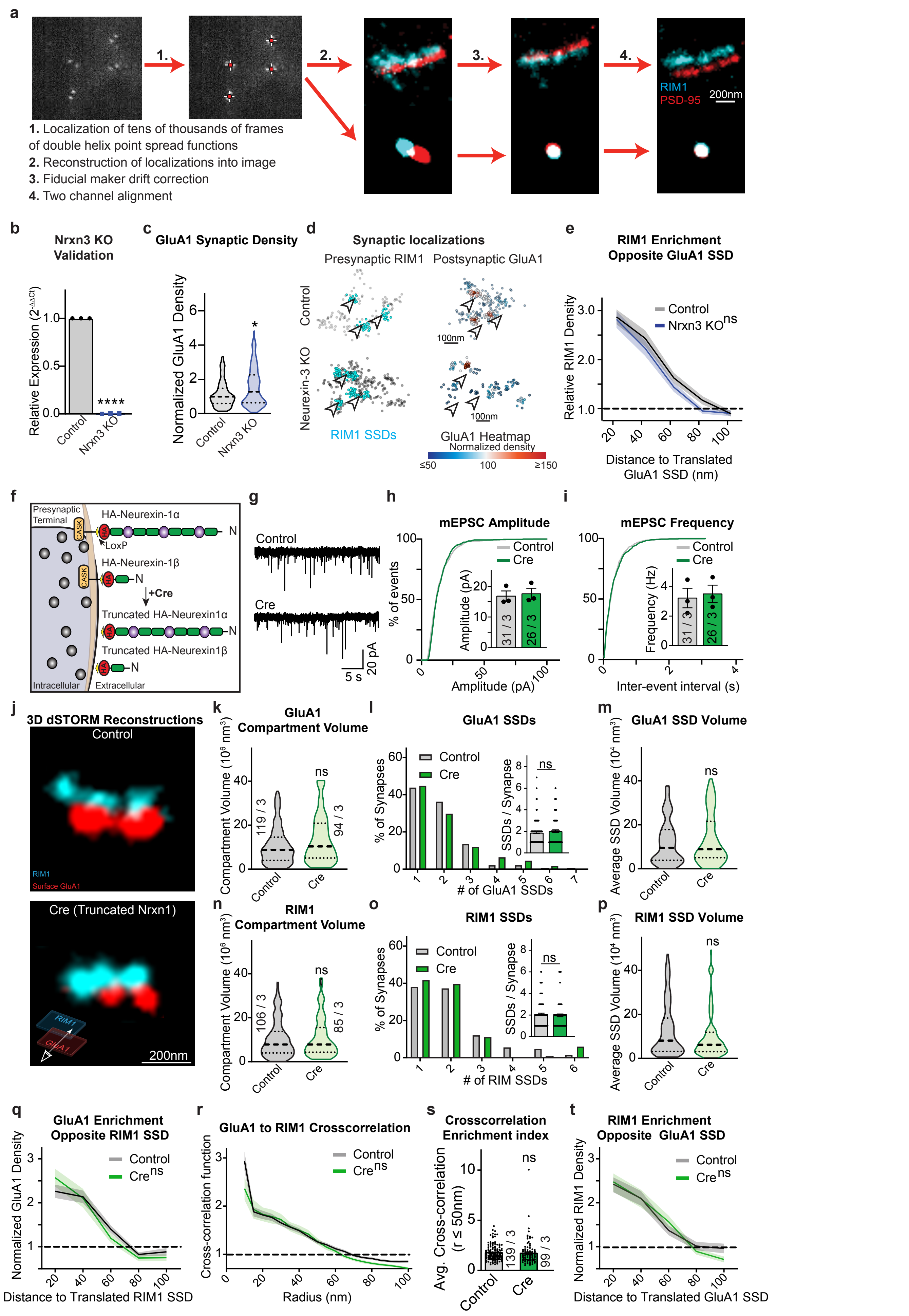
a
1.
2.
3.
4.
RIM1
PSD-95
200nm
1. Localization of tens of thousands of frames of double helix point spread functions
2. Reconstruction of localizations into image
3. Fiducial maker drift correction
4. Two channel alignment
b
Nrxn3 KO Validation
Relative Expression (2^-ΔΔCt)
Control
Nrxn3 KO
****
c
GluA1 Synaptic Density
Normalized GluA1 Density
d
Synaptic localizations
Presynaptic RIM1
Postsynaptic GluA1
Control
Neurexin-3 KO
100nm
RIM1 SSDs
GluA1 Heatmap
Normalized density
≤50
100
≥150
e
RIM1 Enrichment Opposite GluA1 SSD
Relative RIM1 Density
Control
Nrxn3 KO ns
Distance to Translated GluA1 SSD (nm)
f
Presynaptic Terminal
HA-Neurexin-1α
LoxP
HA-Neurexin-1β
+Cre
Truncated HA-Neurexin1α
Truncated HA-Neurexin1β
Intracellular
Extracellular
CASK
g
Control
Cre
5 s
20 pA
h
mEPSC Amplitude
% of events
Amplitude (pA)
Control
Cre
31 / 3
26 / 3
i
mEPSC Frequency
Frequency (Hz)
Inter-event interval (s)
j
3D dSTORM Reconstructions
Control
RIM1
Surface GluA1
Cre (Truncated Nrxn1)
200nm
k
GluA1 Compartment Volume
Compartment Volume (10^6 nm^3)
119 / 3
94 / 3
ns
l
GluA1 SSDs
% of Synapses
SSDs / Synapse
# of GluA1 SSDs
m
GluA1 SSD Volume
Average SSD Volume (10^4 nm^3)
n
RIM1 Compartment Volume
106 / 3
85 / 3
o
RIM1 SSDs
# of RIM SSDs
p
RIM1 SSD Volume
q
GluA1 Enrichment Opposite RIM1 SSD
Normalized GluA1 Density
Distance to Translated RIM1 SSD
r
GluA1 to RIM1 Crosscorrelation
Cross-correlation function
Radius (nm)
s
Crosscorrelation Enrichment index
Avg. Cross-correlation (r ≤ 50nm)
139 / 3
99 / 3
t
RIM1 Enrichment Opposite GluA1 SSD
Normalized RIM1 Density
Distance to Translated GluA1 SSD

Figure S1. *Nrxn1* truncation does not alter GluA1 and RIM1 nanoscale properties or excitatory synaptic transmission in hippocampal culture.

(a) Schematic showing the acquisition of raw data and 3DTRAX analysis workflow as indicated with numbers 1-4. Raw double helix PSFs for localization (left). Reconstructed images (right) with corrections as indicated in figure (top) excitatory synapse (bottom) Tetraspeck bead.
(b) Lentivirus transduction of cre-recombinase into Nrxn3 cKO neurons resulted in nearly undetectable levels of Nrxn3 transcript in dissociated cortical culture, p<0.0001; Unpaired t-test (two-tailed) n=3 independent cultures.
(c) Nrxn3 cKO increases the synaptic density of GluA1, p=0.0129, n=115 control and n=139 Nrxn3 cKO synapses. Significance: Mann Whitney test (two-tailed).
(d) Distribution of synaptic RIM (left) and GluA1 (right) for control (top) and Nrxn3 KO (bottom) neurons. Red and blue clusters indicate RIM1 and GluA1 SSDs, respectively. Arrows indicate enrichment of GluA1 (heatmap) opposite RIM1 SSDs (arrows).
(e) Nrxn3 KO does not affect the transsynaptic enrichment of RIM1 opposite GluA1 SSDs, p=0.1914; n=91 for control and n=85 Nrxn3 KO SSDs. Significance: 2-way repeated measures ANOVA main effect of Nrxn3 KO.
(f) Schematic of 2xHA-Nrxn1 showing location of 2xHA and LoxP site that results in cre-dependent truncation of Nrxn1.
(g-i) Representative mEPSC traces from inactive (control) and active Cre recombinase infected HA-Nrxn1 neurons (g) show no changes in mEPSC amplitude (h; p=0.7742) or mEPSC frequency (i; p=0.7658). Note that cre-mediated truncation of Nrxn1 results in an inactive protein product. Significance: unpaired t-test (two-tailed). n=31 control and 26 Cre neurons from 3 independent experiments.
(j) Representative 3D-dSTORM images of RIM1 (cyan) and GluA1 (red) in control (top) and Cre (truncated Nrxn1) neurons (bottom).
(k-m) Truncation of Nrxn1 did not result in any changes to GluA1 compartment volume (k; p=0.0946), GluA1 SSDs (l; p=0.6425; n=132 control and n=107 cre synapses), or GluA1 SSD volume (m; p=0.4706; n=111 control and 95 cre synapses). Significance: Mann Whitney test (two-tailed).
(n-p) Truncation of Nrxn1 also did not change RIM1 compartment volume (n, p=0.7650), RIM1 SSDs (o; p=0.3934; n=123 control and n=98 cre synapses), or RIM1 SSD volume (p; p=0.0754; n=117 control and n=94 cre synapses). Significance: Mann Whitney test (two-tailed).
(q-t) Nrxn1 truncation did not alter GluA1 / RIM1 transsynaptic alignment including GluA1 enrichment opposite RIM1 SSDs (q; p=0.7806; n=79 control and n=39 cre SSDs), GluA1 to RIM1 cross-correlation (r; p=0.3536; n =139 control and 99 cre synapses), average cross-correlation ≤50nm (s; p=0.0590), or RIM1 enrichment opposite GluA1 SSDs (t; p=0.8294; n=82 control and n=52 cre SSDs). Significance: 2-way repeated measures ANOVA main effect of Cre and Mann Whitney test (two-tailed) as appropriate.
Data from three independent experiments. Number of synapses indicated on graph unless stated in legend. Bar graphs and line graphs: average ± SEM. Violin plots: median ± upper and lower quartiles.

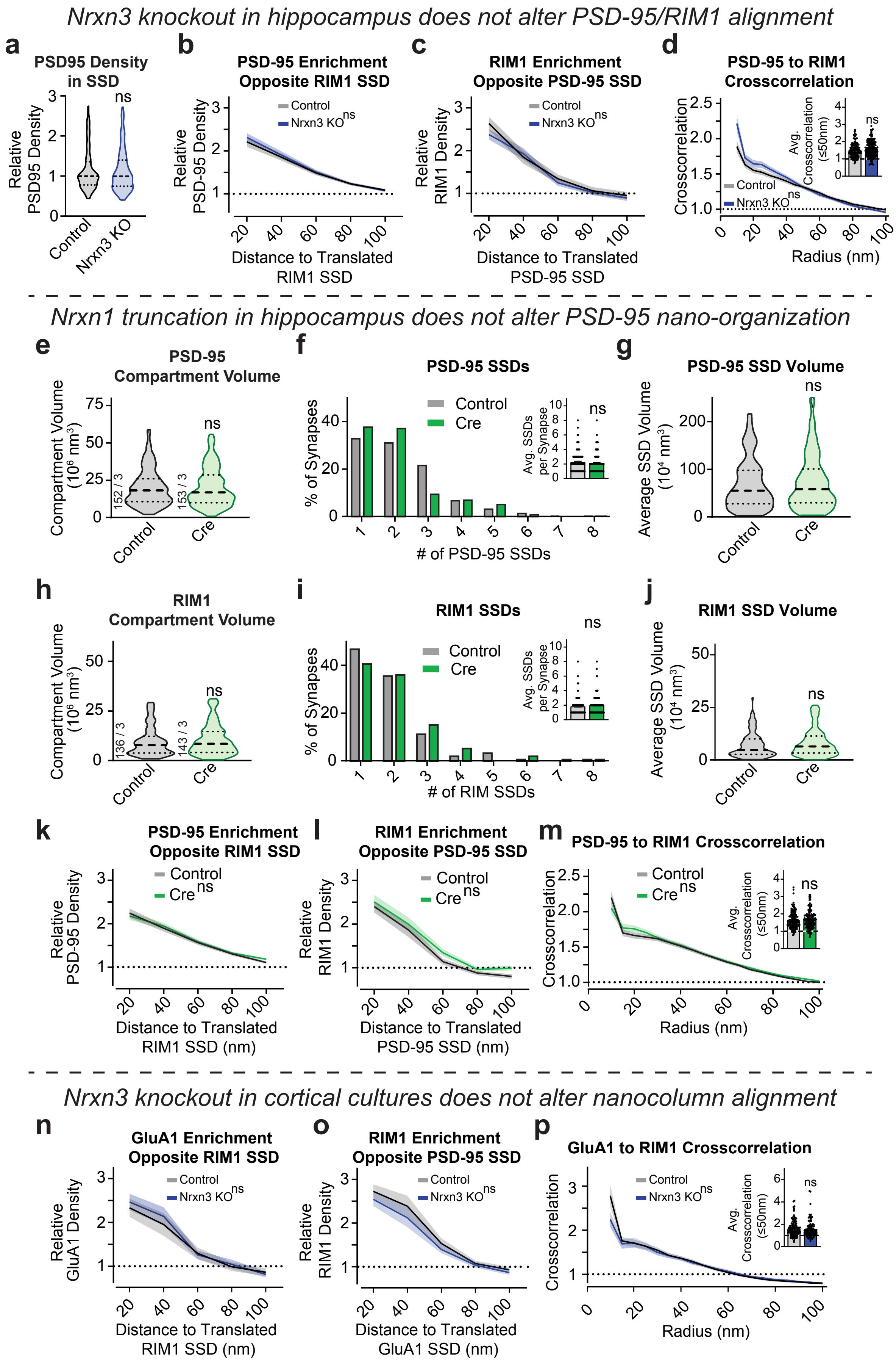
Nrxn3 knockout in hippocampus does not alter PSD-95/RIM1 alignment
a
PSD95 Density in SSD
Relative PSD95 Density
ns
Control
Nrxn3 KO
b
PSD-95 Enrichment Opposite RIM1 SSD
Relative PSD-95 Density
Control
Nrxn3 KO
ns
Distance to Translated RIM1 SSD
c
RIM1 Enrichment Opposite PSD-95 SSD
Relative RIM1 Density
Control
Nrxn3 KO
ns
Distance to Translated PSD-95 SSD
d
PSD-95 to RIM1 Crosscorrelation
Crosscorrelation
Avg. Crosscorrelation (≤50nm)
ns
Control
Nrxn3 KO
ns
Radius (nm)
Nrxn1 truncation in hippocampus does not alter PSD-95 nano-organization
e
PSD-95 Compartment Volume
Compartment Volume (10⁶ nm³)
152 / 3
153 / 3
ns
Control
Cre
f
PSD-95 SSDs
% of Synapses
Control
Cre
Avg. SSDs per Synapse
ns
# of PSD-95 SSDs
g
PSD-95 SSD Volume
Average SSD Volume (10⁴ nm³)
ns
Control
Cre
h
RIM1 Compartment Volume
Compartment Volume (10⁶ nm³)
136 / 3
143 / 3
ns
Control
Cre
i
RIM1 SSDs
% of Synapses
Control
Cre
Avg. SSDs per Synapse
ns
# of RIM SSDs
j
RIM1 SSD Volume
Average SSD Volume (10⁴ nm³)
ns
Control
Cre
k
PSD-95 Enrichment Opposite RIM1 SSD
Relative PSD-95 Density
Control
Cre
ns
Distance to Translated RIM1 SSD (nm)
l
RIM1 Enrichment Opposite PSD-95 SSD
Relative RIM1 Density
Control
Cre
ns
Distance to Translated PSD-95 SSD (nm)
m
PSD-95 to RIM1 Crosscorrelation
Crosscorrelation
Control
Cre
ns
Avg. Crosscorrelation (≤50nm)
ns
Radius (nm)
Nrxn3 knockout in cortical cultures does not alter nanocolumn alignment
n
GluA1 Enrichment Opposite RIM1 SSD
Relative GluA1 Density
Control
Nrxn3 KO
ns
Distance to Translated RIM1 SSD (nm)
o
RIM1 Enrichment Opposite PSD-95 SSD
Relative RIM1 Density
Control
Nrxn3 KO
ns
Distance to Translated GluA1 SSD (nm)
p
GluA1 to RIM1 Crosscorrelation
Crosscorrelation
Control
Nrxn3 KO
ns
Avg. Crosscorrelation (≤50nm)
ns
Radius (nm)

Figure S2. Truncation of *Nrxn1* does not alter PSD-95 nanoscopic properties in hippocampal culture.

(a) Nrxn3 KO does not alter the density of PSD-95 localization in SSDs. Control: n=123 SSDs; Nrxn3 KO: n=138 SSDs from 3 independent experiments. Significance: two-tailed Mann-Whitney. p=0.7444.
(b-d) Nrxn3 KO does not alter PSD-95 / RIM1 transsynaptic alignment including PSD-95 enrichment opposite RIM1 SSDs (b; p=0.6039 n=168 control and n=178 Nrxn3 KO SSDs), RIM1 enrichment opposite PSD-95 SSDs (c; p=0.3815 n=86 control and n=119 Nrxn3 KO SSDs), PSD-95 to RIM1 cross-correlation (d; p=0.3575 n=142 control and n=164 Nrxn3 KO synapses), or average cross-correlation ≤50nm d inset; p=0.1516 n=142 control and n=162 Nrxn3 KO synapses). Significance: 2-way repeated measures ANOVA main effect of Nrxn3 KO and Mann Whitney test (two-tailed) as appropriate.
(e-g) Nrxn1 truncation does not alter PSD-95 compartment volume (e; p=0.9302), PSD-95 SSDs (f; p=0.1530; n=169 control and n=163 cre synapses), or PSD-95 SSD volume (g; p=0.5952; n=155 control and n=147 cre synapses). Significance: Mann Whitney test (two-tailed).
(h-j) Truncation of Nrxn1 does not result in changes to RIM1 compartment volume (h; p=0.6153), RIM1 SSDs (i; p=0.1928; n=152 control and n=153 cre synapses), or RIM1 SSD volume (j; p=0.1227; n=119 control and n=121 cre synapses). Significance: Mann Whitney test.
(k-m) Nrxn1 truncation does not alter measurements of PSD-95–RIM1 transsynaptic alignment including PSD-95 enrichment opposite RIM1 SSDs (k; p=0.7508; n=167 control and n=198 cre SSDs), RIM1 enrichment opposite PSD-95 SSDs (l; p=0.0914; n=100 control n=94 cre SSDs), PSD-95 to RIM1 cross-correlation (m; p=0.5361; n=171 control and n=164 cre synapses) or average cross-correlation ≤50nm (m inset; average cross-correlation ≤50nm, p=0.6400; n=171 control and n=164 cre synapses). Significance: 2-way repeated measures ANOVA main effect of Cre and Mann Whitney test (two-tailed) as appropriate.
(n-p) Nrxn3 KO in cortical neurons does not change nanocolumn alignment including GluA1 enrichment opposite RIM1 SSDs (n, p=0.6386; n=53 control and n=52 Nrxn3 KO SSDs), RIM1 enrichment opposite PSD-95 SSDs (o, p=0.1929; n=60 control and n=61 Nrxn3 KO SSDs), GluA1 to RIM1 cross-correlation (p, p=0.6125; n=130 control and n=116 Nrxn3 KO synapses), or average cross-correlation ≤50nm (p inset, p=0.2693; n=130 control and n=116 Nrxn3 KO synapses). Significance: 2-way repeated measures ANOVA main effect of Nrxn3 KO and Mann Whitney test (two-tailed) as appropriate.
Data from three independent experiments. Number of synapses indicated on graph unless stated in legend. Bar graphs and line graphs: average ± SEM. Violin plots: median ± upper and lower quartiles.

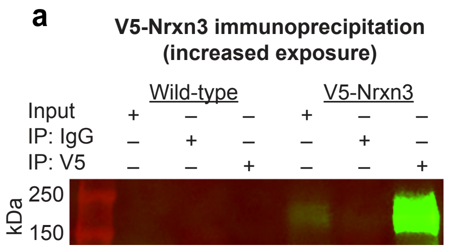

V5-Nrxn3 does not affect pan-Nrxn nanoscale organization at Homer1+ synapses

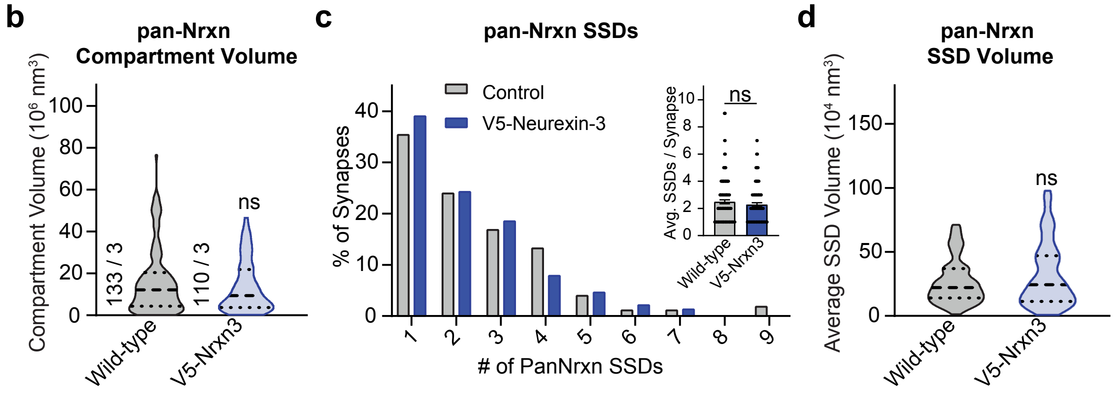

Figure S3. The nanoscale properties of pan-Nrxns are unaltered in *V5-Nrxn3* KI cultures.
(a) Over-exposed image of V5-Nrxn3 immunoblot to show the presence of V5 immunoreactivity in V5-Nrxn3$^{KI/KI}$ sample but not wildtype.
(b-d) Endogenous expression of V5-Nrxn3 does not alter the nanoscale properties of pan-Nrxns including pan-Nrxn compartment volume (b; p=0.3714), number of SSDs (c; p=0.4028; n=140 wild-type and n=122 V5-Nrxn3 synapses), or SSD volume (d; p=0.5030; n=125 wild-type and n=109 V5-Nrxn3 synapses). Significance: Mann Whitney test (two-tailed). Data from three independent experiments.
Number of synapses indicated on graph unless stated in legend. Bar graphs and line graphs: average ± SEM. Violin plots: median ± upper and lower quartiles.

V5-Nrxn3 does not affect the number or alignment of PSD-95 or RIM1 SSDs

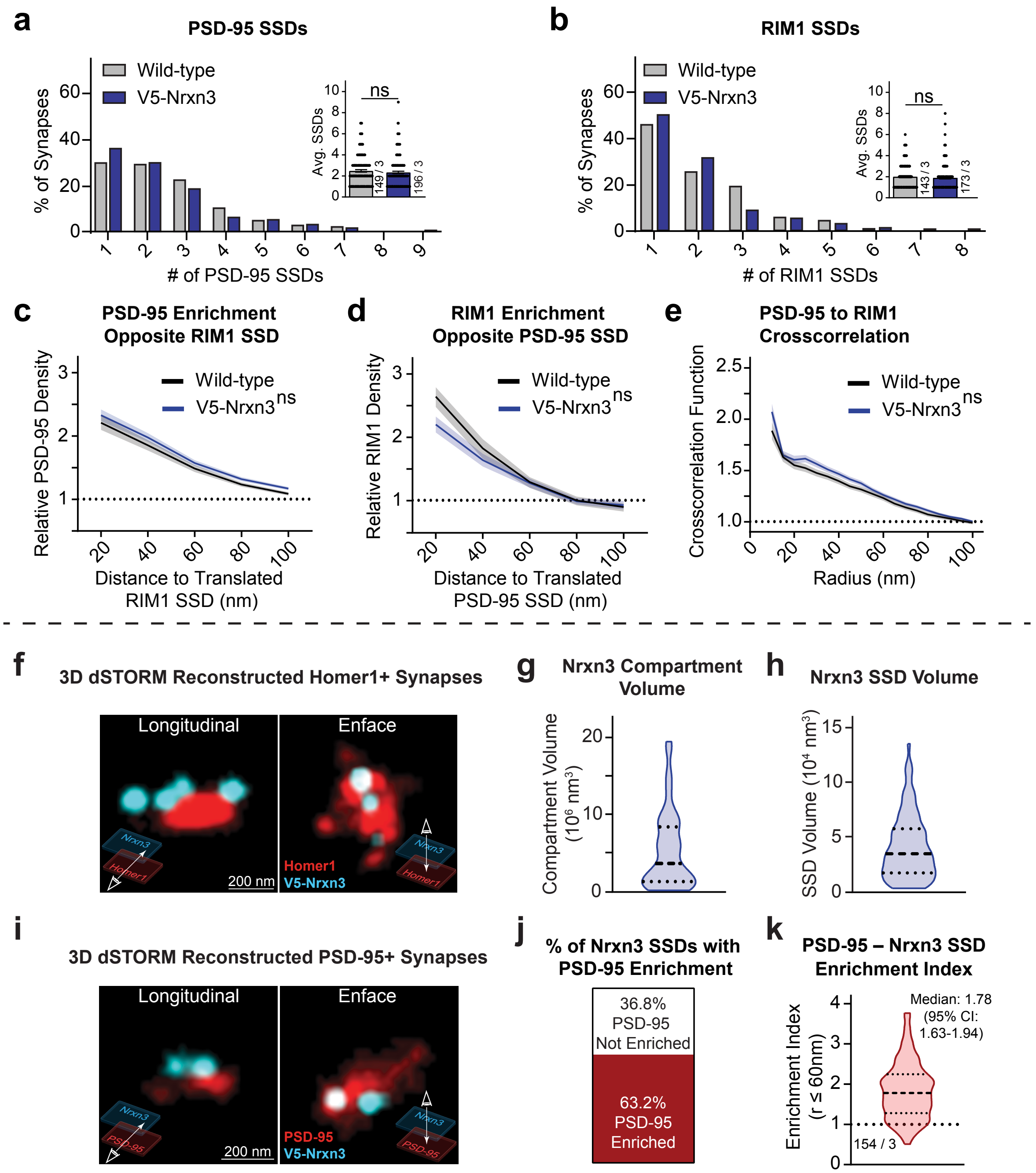

Figure S4. V5-Nrxn3 does not alter excitatory transsynaptic alignment of PSD-95 and RIM1 and is enriched in synaptic nanocolumns.

(a-b) Endogenous V5-Nrxn3 expression does not alter the average number of PSD-95 SSDs (a; p=0.1877) or RIM1 SSDs (b; p=0.2209; n=143 wild-type and n=173 V5-Nrxn3 synapses). Significance: Mann Whitney test (two-tailed).

(c-e) V5-Nrxn3 does not change transsynaptic alignment of PSD-95 and RIM1 including PSD-95 enrichment opposite RIM1 SSDs (c; p=0.0843; n=168 wild-type and n=201 V5-Nrxn3 SSDs), RIM1 enrichment opposite PSD-95 SSDs (d; p=0.1056; n=85 wild-type and n=118 V5-Nrxn3 SSDs), and PSD-95 to RIM1 cross-correlation (e; p=0.1110; n=142 wild-type and n=195 V5-Nrxn3 synapses). Significance: 2-way repeated measures ANOVA main effect of mouse line.

(f) Representative 3D-dSTORM reconstructions of V5-Nrxn3 (cyan) and Homer1 (red) in the longitudinal (left) and enface (right) orientations.

(g) Violin plot of V5-Nrxn3 compartment volume at Homer1+ synapses. n=93 synapses.

(h) Violin plot of average V5-Nrxn3 SSD volume at Homer1+ synapses. n=98 synapses.

(i) Representative 3D-STORM reconstructions of V5-Nrxn3 (cyan) and PSD-95 (red) in the longitudinal (left) and en face (right) orientations.

(j) Stacked bar graph showing that 63.2% of Nrxn3 SSDs had PSD-95 enrichment index of >1.25. n=266 SSDs.

(k) PSD-95 enrichment at distances of 20-60nm from the translated Nrxn3 SSD.

Data from three independent experiments. Number of synapses indicated on graph unless stated in legend. Bar graphs and line graphs: average ± SEM. Violin plots: median ± upper and lower quartiles.

HA-Nrxn1 does not affect the number or alignment of PSD-95 or RIM1 SSDs

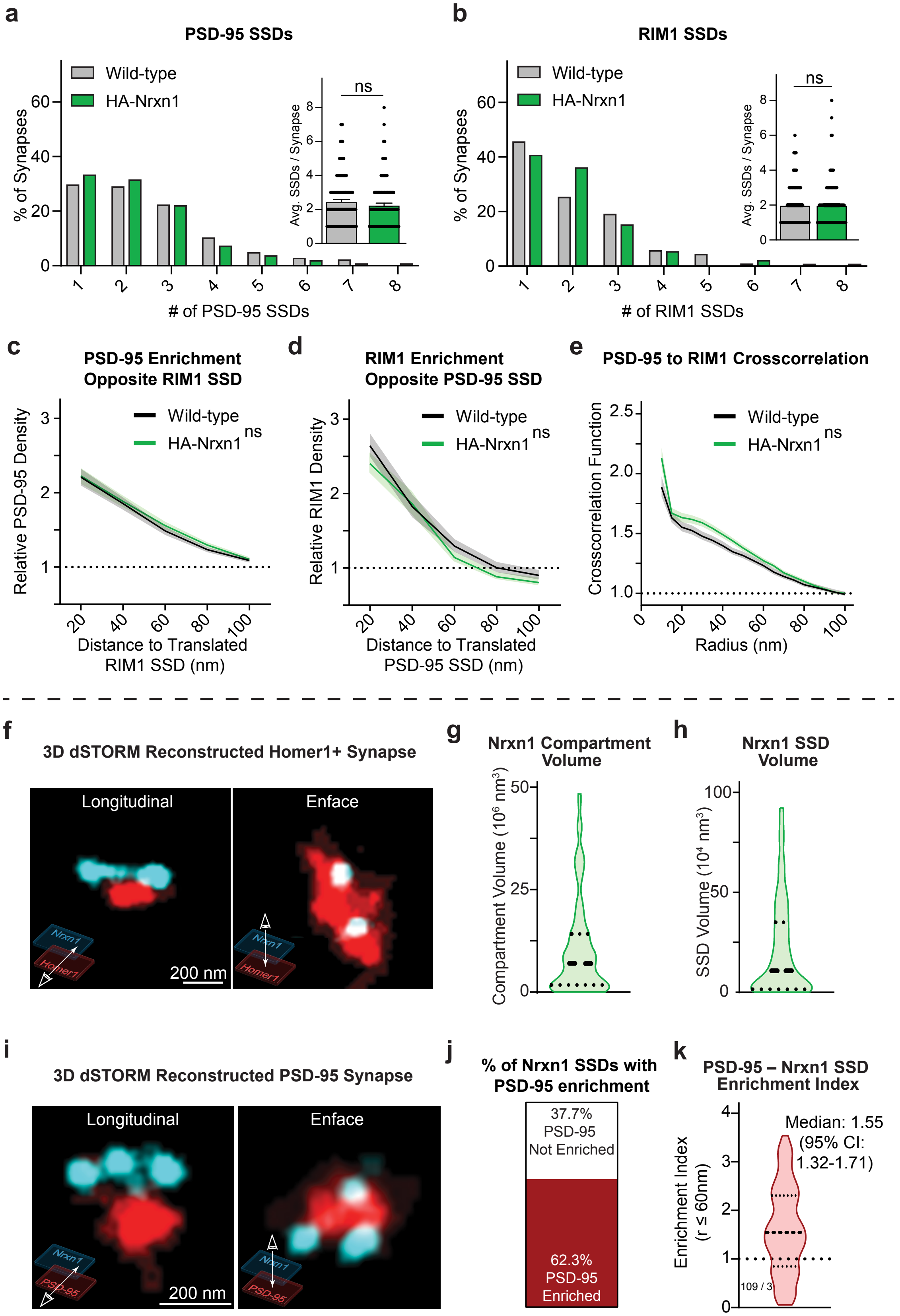

Figure S5. HA-Nrxn1 does not alter excitatory synaptic nanocolumns.
(a-b) Endogenous expression of HA-Nrxn1 does not alter the number of PSD-95 SSDs (a; p=0.2201) or RIM1 SSDs (b; p=0.9505; n=143 wild-type and n=153 V5-Nrxn3 synapses). Significance: Mann Whitney test (two-tailed).
(c-e) HA-Nrxn1 does not alter the transsynaptic alignment of PSD-95 enrichment opposite RIM1 SSDs (c; p=0.5280; n=168 wild-type and n=166 HA-Nrxn1 SSDs), RIM1 enrichment opposite PSD-95 SSDs (d; p=0.1594; n=85 wild-type and n=100 HA-Nrxn1 SSDs), or PSD-95 to RIM1 cross-correlation (e; p=0.0648; n=142 control and n=168 HA-Nrxn1 synapses). Significance: 2-way repeated measures ANOVA main effect of mouse line.
(f) Representative 3D-dSTORM reconstructions of HA-Nrxn1 (cyan) and Homer1 (red) in the longitudinal (left) and enface (right) orientations.
(g-h) Violin plots of HA-Nrxn1 compartment volume (g; n=147 synapses) and HA-Nrxn1 SSD volume (h; n=148 synapses) at Homer1+ synapses.
(i) Representative 3D-dSTORM reconstructions of HA-Nrxn1 (cyan) and PSD-95 (red) in the longitudinal (left) and enface (right) orientations.
(j) Stacked bar graph showing that 62.3% of Nrxn1 SSDs had PSD-95 enrichment index of >1.25. n=151 SSDs.
(k) PSD-95 enrichment index (20-60nm) from the translated Nrxn1 SSD.
Data from three independent experiments. Number of synapses indicated on graph unless stated in legend. Bar graphs and line graphs: average ± SEM. Violin plots: median ± upper and lower quartiles.

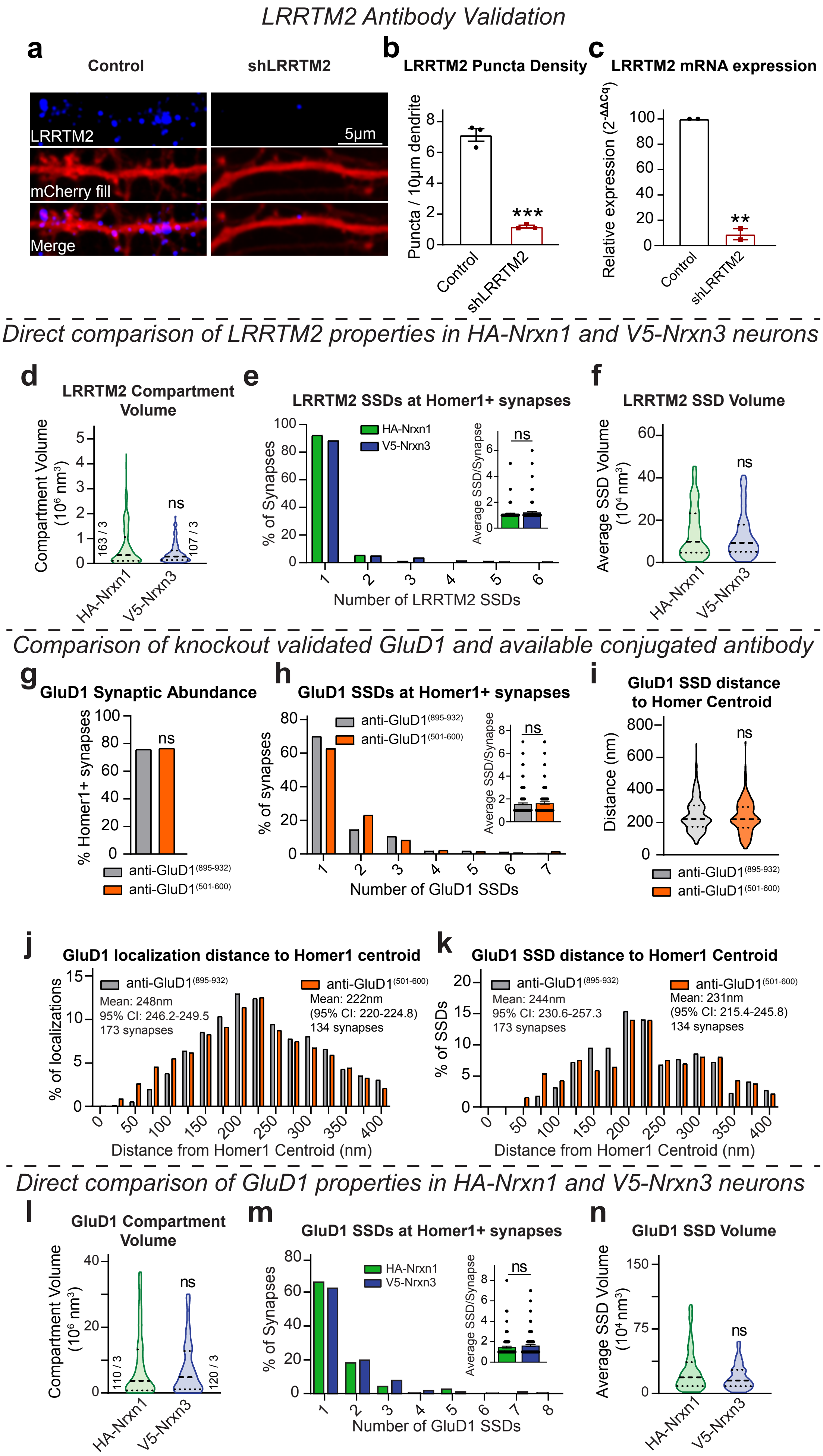
LRRTM2 Antibody Validation
a
Control
shLRRTM2
LRRTM2
5µm
mCherry fill
Merge
b
LRRTM2 Puncta Density
Puncta / 10µm dendrite
***
Control
shLRRTM2
c
LRRTM2 mRNA expression
Relative expression ($2^{-\Delta\Delta Cq}$)
**
Control
shLRRTM2
Direct comparison of LRRTM2 properties in HA-Nrxn1 and V5-Nrxn3 neurons
d
LRRTM2 Compartment Volume
Compartment Volume (10^6 nm^3)
163 / 3
107 / 3
ns
HA-Nrxn1
V5-Nrxn3
e
LRRTM2 SSDs at Homer1+ synapses
% of Synapses
HA-Nrxn1
V5-Nrxn3
Average SSD/Synapse
ns
Number of LRRTM2 SSDs
f
LRRTM2 SSD Volume
Average SSD Volume (10^4 nm^3)
ns
HA-Nrxn1
V5-Nrxn3
Comparison of knockout validated GluD1 and available conjugated antibody
g
GluD1 Synaptic Abundance
% Homer1+ synapses
ns
anti-GluD1$^{(895\text{-}932)}$
anti-GluD1$^{(501\text{-}600)}$
h
GluD1 SSDs at Homer1+ synapses
% of synapses
anti-GluD1$^{(895\text{-}932)}$
anti-GluD1$^{(501\text{-}600)}$
Average SSD/Synapse
ns
Number of GluD1 SSDs
i
GluD1 SSD distance to Homer Centroid
Distance (nm)
ns
anti-GluD1$^{(895\text{-}932)}$
anti-GluD1$^{(501\text{-}600)}$
j
GluD1 localization distance to Homer1 centroid
% of localizations
anti-GluD1$^{(895\text{-}932)}$
Mean: 248nm
95% CI: 246.2-249.5
173 synapses
anti-GluD1$^{(501\text{-}600)}$
Mean: 222nm
(95% CI: 220-224.8)
134 synapses
Distance from Homer1 Centroid (nm)
k
GluD1 SSD distance to Homer1 Centroid
% of SSDs
anti-GluD1$^{(895\text{-}932)}$
Mean: 244nm
95% CI: 230.6-257.3
173 synapses
anti-GluD1$^{(501\text{-}600)}$
Mean: 231nm
(95% CI: 215.4-245.8)
134 synapses
Distance from Homer1 Centroid (nm)
Direct comparison of GluD1 properties in HA-Nrxn1 and V5-Nrxn3 neurons
l
GluD1 Compartment Volume
Compartment Volume (10^6 nm^3)
110 / 3
120 / 3
ns
HA-Nrxn1
V5-Nrxn3
m
GluD1 SSDs at Homer1+ synapses
% of Synapses
HA-Nrxn1
V5-Nrxn3
Average SSD/Synapse
ns
Number of GluD1 SSDs
n
GluD1 SSD Volume
Average SSD Volume (10^4 nm^3)
ns
HA-Nrxn1
V5-Nrxn3

Figure S6. Validation of surface LRRTM2 and cytoplasmic GluD1 antibodies.
(a) Representative images of Control (mock infected) and shLRRTM2 neurons stained with an AF647 conjugated anti-LRRTM2 antibody.
(b) shRNA knockdown of LRRTM2 reduced puncta density p=0.0001. Unpaired t-test (two-tailed) n=3 independent cultures.
(c) Quantitative mRNA measurements following shRNA knockdown of endogenous LRRTM2 shows reduced relative expression of *LRRTM2* p=0.0023. Unpaired t-test (two-tailed) n=3 independent cultures.
(d-f) Comparison of LRRTM2 nanoscale properties in HA-Nrxn1 and V5-Nrxn3 neurons show no differences in LRRTM2 compartment volume (d; p=0.3033), number of SSDs (e; p=0.2108; n=182 HA-Nrxn1 and n=138 V5-Nrxn3 synapses), or average SSD volume (f; p=0.6241; n=148 HA-Nrxn1 and n=101 V5-Nrxn3 synapses). Significance: Mann Whitney test (two-tailed).
(g-h) GluD1 antibodies targeting amino acids 895-932 (anti-GluD1$^{895\text{-}932}$) and 501-600 (anti-GluD1$^{501\text{-}600}$) show no differences in the proportion of Homer1+ synapses containing GluD1 (g; p=0.8773; n=232 anti-GluD1$^{895\text{-}932}$ synapses and n=196 GluD1$^{501\text{-}600}$ synapses) or the average number of GluD1 SSDs (h; p=0.2880; n=173 anti-GluD1$^{895\text{-}932}$ synapses and n=134 GluD1$^{501\text{-}600}$ synapses). Significance: χ^2 test and Mann Whitney test respectively (two-tailed).
(i-k) anti-GluD1$^{895\text{-}932}$ and anti-GluD1$^{501\text{-}600}$ show no significant differences in the median distance from GluD1 SSDs to the Homer1 centroid (i; p=0.3376; n=238 anti-GluD1$^{895\text{-}932}$ SSDs and n=192 GluD1$^{501\text{-}600}$ SSDs) and show similar radial distributions of GluD1 localizations (j) and GluD1 SSDs (k) at Homer1+ synapses with mean and 95% confidence interval of a Poisson distribution indicated on graph. Significance: Mann Whitney test (two-tailed).
(l-n) The nanoscale properties of anti-GluD1$^{501\text{-}600}$ exhibit no differences in HA-Nrxn1 and V5-Nrxn3 neurons. GluD1 compartment volume (l; p=0.7532), number of SSDs (m; p=0.0984; n=129 HA-Nrxn1 synapses and n=134 V5-Nrxn3 synapses), average SSD volume (n; p=0.1389; n=88 HA-Nrxn1 synapses and n=111 V5-Nrxn3 synapses). Significance: Mann Whitney test (two-tailed).
Data from three independent experiments. Number of synapses indicated on graph unless stated in legend. Bar graphs and line graphs: average ± SEM. Violin plots: median ± upper and lower quartiles.

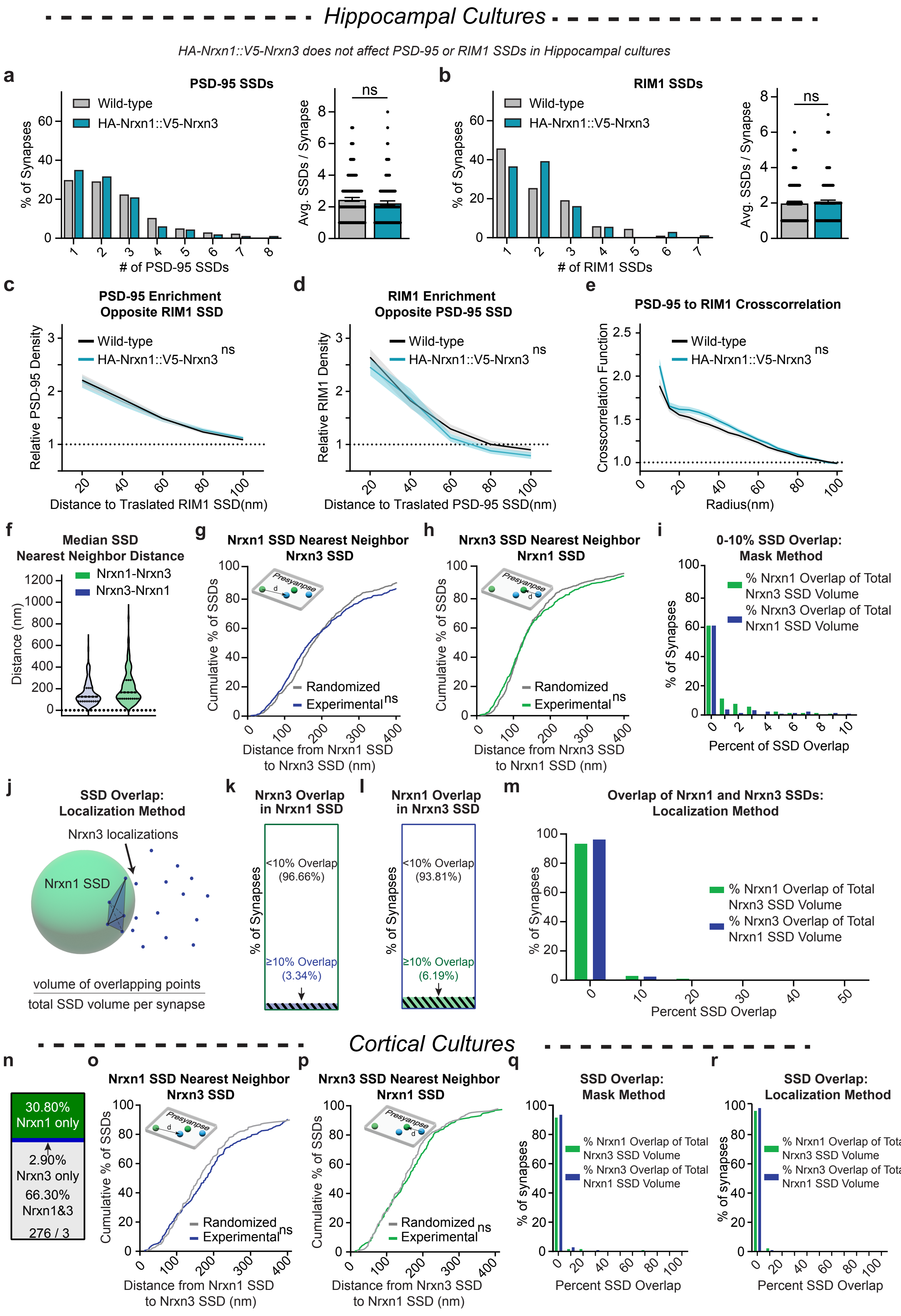

Hippocampal Cultures
HA-Nrxn1::V5-Nrxn3 does not affect PSD-95 or RIM1 SSDs in Hippocampal cultures
a
PSD-95 SSDs
Wild-type
HA-Nrxn1::V5-Nrxn3
% of Synapses
# of PSD-95 SSDs
ns
Avg. SSDs / Synapse
b
RIM1 SSDs
# of RIM1 SSDs
c
PSD-95 Enrichment Opposite RIM1 SSD
Relative PSD-95 Density
Distance to Traslated RIM1 SSD(nm)
d
RIM1 Enrichment Opposite PSD-95 SSD
Relative RIM1 Density
Distance to Traslated PSD-95 SSD(nm)
e
PSD-95 to RIM1 Crosscorrelation
Crosscorrelation Function
Radius(nm)
f
Median SSD Nearest Neighbor Distance
Nrxn1-Nrxn3
Nrxn3-Nrxn1
Distance (nm)
g
Nrxn1 SSD Nearest Neighbor Nrxn3 SSD
Presyanpse
Randomized
Experimental
Cumulative % of SSDs
Distance from Nrxn1 SSD to Nrxn3 SSD (nm)
h
Nrxn3 SSD Nearest Neighbor Nrxn1 SSD
Distance from Nrxn3 SSD to Nrxn1 SSD (nm)
i
0-10% SSD Overlap: Mask Method
% Nrxn1 Overlap of Total Nrxn3 SSD Volume
% Nrxn3 Overlap of Total Nrxn1 SSD Volume
Percent of SSD Overlap
j
SSD Overlap: Localization Method
Nrxn3 localizations
Nrxn1 SSD
volume of overlapping points
total SSD volume per synapse
k
Nrxn3 Overlap in Nrxn1 SSD
<10% Overlap (96.66%)
≥10% Overlap (3.34%)
l
Nrxn1 Overlap in Nrxn3 SSD
<10% Overlap (93.81%)
≥10% Overlap (6.19%)
m
Overlap of Nrxn1 and Nrxn3 SSDs: Localization Method
Percent SSD Overlap
Cortical Cultures
n
30.80% Nrxn1 only
2.90% Nrxn3 only
66.30% Nrxn1&3
276 / 3
o
Nrxn1 SSD Nearest Neighbor Nrxn3 SSD
p
Nrxn3 SSD Nearest Neighbor Nrxn1 SSD
q
SSD Overlap: Mask Method
% of synapses
r
SSD Overlap: Localization Method

Figure S7. Excitatory synaptic nanocolumns are maintained in *HA-Nrxn1::V5-Nrxn3* synapses and Nrxn1 and Nrxn3 are spatially distinct in cortical culture.
(a-b) The nanoscale properties of excitatory synapses are not impacted in neurons that endogenously express both HA-Nrxn1 and V5-Nrxn3. The number of PSD-95 SSDs (a; p=0.1702) or RIM1 SSDs (b; p=0.5552; n=143 wild-type and n=113 HA-Nrxn1::V5-Nrxn3 synapses from two independent experiments). Significance: Mann Whitney test (two-tailed).
(c-e) Neurons from HA-Nrxn1::V5-Nrxn3 animals do not show any differences from wild-type neurons in measurements of transsynaptic alignment including PSD-95 enrichment opposite RIM1 SSDs (c; p=0.9487; n=168 wild-type and n=136 HA-Nrxn1::V5-Nrxn3 synapses), RIM1 enrichment opposite PSD-95 SSDs (d; p=0.2072; n=85 wild-type and n=76 HA-Nrxn1::V5-Nrxn3 synapses), or PSD-95 to RIM1 cross-correlation (e; p=0.1150 n=142 wild-type and n=151 HA-Nrxn1::V5-Nrxn3 synapses). Significance 2-way repeated measures ANOVA main effect of mouse line. Data from 3 independent experiments for wild-type and 2 independent experiments for HA-Nrxn1::V5-Nrxn3.
(f) Violin plots of nearest neighbor distances between Nrxn1 and Nrxn3 SSDs. n=345 Nrxn3 and n=541 Nrxn1 SSDs.
(g-h) Nearest neighbor distances of Nrxn1 SSDs to Nrxn3 SSDs (g) or Nrxn3 SSDs to Nrxn1 SSDs in hippocampal synapses (h). Relative to randomized values, Nrxn1 to Nrxn3 nearest neighbor distances, p=0.0767 (541 experimental and randomized SSDs); Nrxn3 to Nrxn1 nearest neighbor distances, p=0.4845 (345 experimental and randomized SSDs). Significance: Kolmogorov-Smirnov test.
(i) Histogram of 0-10% bin from Figure 7K showing the majority of these synapses have less than 1% overlap; n=155 synapses for Nrxn1 overlap and n=187 synapses for Nrxn3 overlap.
(j) Schematic of the overlapping localizations method.
(k-l) Stacked bar graphs of Nrxn3 localization overlap with Nrxn1 SSDs (k) and Nrxn1 localization overlap with Nrxn3 SSDs (l). n=210 synapses.
(m) Frequency of percent overlap as determined by the overlapping localizations method. n=210 synapses.
(n) Stacked bar graph showing the percentage of Homer1+ and Nrxn+ cortical synapses that contained HA-Nrxn1 only (top; green), V5-Nrxn3 only (middle; blue), or both HA-Nrxn1 and Nrxn3 (bottom; white).
(o-p) Same as (g-h) except in cortical synapses. The observed nearest neighbor distance between HA-Nrxn1 and V5-Nrxn3 was not closer than expected by chance when measuring nearest neighbor distances from HA-Nrxn1 to V5-Nrxn3 SSDs (o; p=0.3763; n=217 randomized and experimental SSDs) and vice versa (p; p=0.5243, n=194 randomized and experimental SSDs). Significance: Kolmogorov-Smirnov test.
(q) Histogram of percent overlap of HA-Nrxn1 and V5-Nrxn3 SSDs in cortical cultures using the mask method. n=149 synapses.
(r) Histogram showing the observed percent overlap of HA-Nrxn1 and V5-Nrxn3 SSDs using the points method. n=149 synapses.
Data from three independent experiments unless otherwise noted. Number of synapses indicated on graph unless stated in legend. Bar graphs and line graphs: average ± SEM. Violin plots: median ± upper and lower quartiles.

Table S1. Summary of Nanoscale properties

Related to Figures 1 and S1								
	Control				Nrxn3 KO			
**Measure**	**Mean**	**Lower Quartile**	**Median**	**Upper Quartile**	**Mean**	**Lower Quartile**	**Median**	**Upper Quartile**
GluA1 Compartment Volume (10^6 nm^3)	17.49	5.247	10.69	21.85	11.86	3.4	8.813	16.33
GluA1 SSDs (avg. SSDs/synapse)	1.828	1	2	2	1.51	1	1	2
Avg. GluA1 SSD Volume (10^4 nm^3)	12.4	4.312	9.508	17.69	14.22	4.07	9.424	18.12
RIM1 Compartment Volume (10^6 nm^3)	21.26	7.563	16.52	31.42	11.87	4.656	9.187	17.56
RIM1 SSDs (avg. SSDs/synapse)	2.194	1	2	3	2.054	1	2	3
Avg. RIM1 SSD Volume (10^4 nm^3)	18.03	5.508	14.08	25	11.05	3.724	8.842	16.35
GluA1 Erichment Index (% increase in relative density ≤60nm)	102.9%	21.90%	90.80%	172.50%	77.2%	22.30%	63.30%	116.40%
Avg. Crosscorrelation<br><br>Enrichment Index (% increase in probability ≤50nm)	82.50%	17.20%	63.00%	140.40%	59%	16.50%	48.30%	95.10%
GluA1 relative density in SSDs (normalized)	1.376	0.7262	1	1.791	1.121	0.4727	0.808	1.581
**Related to Figure 2 and S2**								

	**Control**				**Nrxn3 KO**			
**Measure**	**Mean**	**Lower Quartile**	**Median**	**Upper Quartile**	**Mean**	**Lower Quartile**	**Median**	**Upper Quartile**
PSD-95 Compartment Volume (10^6 nm^3)	27.89	12.08	23.35	38	14.69	6.718	12.11	20.92
PSD-95 SSDs (avg. SSDs/synapse)	2.477	1	2	3	1.933	1	1	2
Avg. PSD-95 SSD Volume (10^4 nm^3)	39.62	14.53	32.31	57.6	40.58	15.35	30.52	57.24
RIM1 Compartment Volume (10^6 nm^3)	17.05	6.022	12.89	25.96	8.428	2.815	6.327	12.78
RIM1 SSDs (avg. SSDs/synapse)	2	1	2	3	1.791	1	1	2
Avg. RIM1 SSD Volume (10^4 nm^3)	12.43	4.626	9.725	17.71	8.141	2.982	6.538	11.81
**Related to Figure 5 and S4**								

	**Experimental**				**Randomized**			
**Measure**	**Mean**	**Lower Quartile**	**Median**	**Upper Quartile**	**Mean**	**Lower Quartile**	**Median**	**Upper Quartile**
Nrxn3 SSD nearest neighbor PSD-95 SSD (nm)	130.1	86.99	119.9	164.9	152.7	94.28	141.8	195.1
PSD-95 SSD nearest neighbor Nrxn3 SSD	162.9	103.9	146.3	208.5	187.1	118.2	173.3	239.8
PSD-95 Enrichment Index (% increase in relative density)	82.60%	28.00%	77.80%	124.70%	N/A			
**Related to Figure 6 and S5**								

	Experimental				Randomized			
**Measure**	**Mean**	**Lower Quartile**	**Median**	**Upper Quartile**	**Mean**	**Lower Quartile**	**Median**	**Upper Quartile**
Nrxn1 SSD nearest neighbor PSD-95 SSD (nm)	205.2	116.8	192.1	272	227.7	134.3	205.1	298.3
PSD-95 SSD nearest neighbor Nrxn1 SSD	241	140.1	214.6	311.6	267.1	157.4	242.2	356.6
Avg. PSD-95 enrichment opposite Nrxn1 SSD (% increase in relative density)	59.40%	-15.60%	54.60%	130.30%	N/A			
**Related to Figure 7**								
	**Nrxn1**				**Nrxn3**			
**Measure**	**Mean**	**Lower Quartile**	**Median**	**Upper Quartile**	**Mean**	**Lower Quartile**	**Median**	**Upper Quartile**
LRRTM2 SSD nearest neighbor Nrxn SSD (nm)	164	116.1	147.5	200.6	133.1	71.9	121.7	185.9
GluD1 SSD nearest neighbor Nrxn SSD (nm)	159.5	105.1	155.2	199	201.4	130	184	263.1
**Related to Figure 8**								
	**Nrxn1**				**Nrxn3**			
**Measure**	**Mean**	**Lower Quartile**	**Median**	**Upper Quartile**	**Mean**	**Lower Quartile**	**Median**	**Upper Quartile**
RIM1 SSD nearest neighbor Nrxn SSD (nm)	229	126.8	199.3	307.1	167.3	93.48	146.5	215.8
Nrxn SSD nearest neighbor RIM1 SSD (nm)	226.1	126.6	202.7	307	148.6	83.84	131.6	191.6
