# Supplementary material for: Neurexin-3 subsynaptic densities are spatially distinct from Neurexin-1 and essential for excitatory synapse nanoscale organization in the hippocampus
Source: Nat Commun. 2023 Aug 5;14:4706. doi: 10.1038/s41467-023-40419-2 (PMC10404257; doi:10.1038/s41467-023-40419-2)
Supplement: Supplementary file 3 — Reporting Summary [file 41467_2023_40419_MOESM3_ESM.pdf]

nature portfolio

Corresponding author(s): Jason Aoto

Last updated by author(s): Jul 11, 2023

Reporting Summary

Nature Portfolio wishes to improve the reproducibility of the work that we publish. This form provides structure for consistency and transparency in reporting. For further information on Nature Portfolio policies, see our Editorial Policies and the Editorial Policy Checklist.

Statistics

For all statistical analyses, confirm that the following items are present in the figure legend, table legend, main text, or Methods section.

n/a	Confirmed	
☐	☒	The exact sample size (*n*) for each experimental group/condition, given as a discrete number and unit of measurement
☐	☒	A statement on whether measurements were taken from distinct samples or whether the same sample was measured repeatedly
☐	☒	The statistical test(s) used AND whether they are one- or two-sided<br>*Only common tests should be described solely by name; describe more complex techniques in the Methods section.*
☐	☒	A description of all covariates tested
☐	☒	A description of any assumptions or corrections, such as tests of normality and adjustment for multiple comparisons
☐	☒	A full description of the statistical parameters including central tendency (e.g. means) or other basic estimates (e.g. regression coefficient) AND variation (e.g. standard deviation) or associated estimates of uncertainty (e.g. confidence intervals)
☐	☒	For null hypothesis testing, the test statistic (e.g. *F*, *t*, *r*) with confidence intervals, effect sizes, degrees of freedom and *P* value noted<br>*Give P values as exact values whenever suitable.*
☒	☐	For Bayesian analysis, information on the choice of priors and Markov chain Monte Carlo settings
☒	☐	For hierarchical and complex designs, identification of the appropriate level for tests and full reporting of outcomes
☒	☐	Estimates of effect sizes (e.g. Cohen's *d*, Pearson's *r*), indicating how they were calculated

Our web collection on statistics for biologists contains articles on many of the points above.

Software and code

Policy information about availability of computer code

Data collection	All Double Helix PSF localization, fiducial marker drift correction, and two channel alignment was completed using Double Helix Optics 3DTRAX® Software (version 3.0b2).
Data analysis	R Version 4.0.3, Matlab Version R2021a, Clampfit 10.7, ImageJ 1.53t, Image Studio 5.2, and GraphPad Prism 9.1.2 were used for analysis. Synapses were identified using a custom R script utilizing a non-biased DBScan method with an epsilon of 100nm and minimum points of 10 as has been widely been used in the past. All synapses were then validated manually to ensure that each potential synapse cluster contained only one synapse, that the localizations appeared synaptic (roughly a disk shape), and that the cluster was not a Tetraspeck bead. Identified synapses were then reclustered using a mean minimal distance method cutoff of 4 to remove localizations or additional synapses that were far from the synapse center using a custom R script. This reclustered synapse data was then subjected to final analysis using MATLAB scripts from Chen et al.19 methods to calculate subsynaptic properties of the proteins of interest. For GluA1, PSD-95, RIM1, and GRID1 nanocluster_detection_3D thresholds of T = 2.5, cutoff = 100nm, and density standard deviation cutoff of 4 was used. For V5-Nrxn3, HA-Nrxn1, and LRRTM2 cutoffs of T = 2, cutoff = 100nm, and density standard deviation cutoff of 1.5 was used. Synapses with more than 5 localizations for V5-Nrxn3, HA-Nrxn1, LRRTM2, and GRID1 were considered positive for that protein and at least 1 SSD.<br>The alphavol function19 was used to determine the volume of the synapse and SSDs. For compartment volume, the input to alphavol was all the synaptic localizations for each protein. To calculate average SSD volume, the alphavol of each SSD in a synapse was calculated and then averaged for that synapse. To determine the relative density of synaptic proteins in SSDs, the density of localizations in SSDs was divided by the density of localizations in the entire synaptic compartment and normalized to the enrichment control synapses.<br>For SSD nearest neighbor analysis, the SSD centers were first extracted from the nanocluster_detection_3D analysis. For experimental conditions, the MATLAB knnsearch function was used on the set of SSDs in both channels. To generate a random selection of SSD centers, the localizations of one channel were randomized to an even distribution within the compartment volume using the get_cluster_randomized function from Chen et al. 2020 Methods and then random SSD centers were picked from this distribution. For each synapse, the number of

selected randomized SSDs was equal to the number of experimental SSDs.
To determine the radial distribution of localizations SSDs at synapses, the pdist2 function was used as has been used in the past. Briefly for comparisons of presynaptic proteins to Homer1 centroid, either localization coordinates or SSD centers were assayed for their distance to the centroid (mean point) of Homer1.
For quantification of Nrxn SSD overlap, two methods were utilized to validate the results. The first method was to quantity the overlap the masks generated by SSDs in each channel. This method is more likely to overestimate the amount of overlap because the polygon mask of the SSD points may include volume where no localizations are present. Briefly, the Matlab function delaunayTriangulation was used to generate polygons of each SSD and a mask was generated of SSDs for Nrxn1 and Nrxn3 by generating a mesh with voxel size of 10nm and testing whether each voxel of the mesh contained a portion of the SSD. The masks for Nrxn1 and Nrxn3 were then multiplied to obtain a mask of only the overlapping points. The volume of the overlapping mask was determined by summing the number of overlap mask voxels and the volume of each channel was calculated as the sum of their individual masks. For the second method, the SSD overlap was determined as the volume of SSD points in channel 1 that reside within the SSD volume of channel 2. Briefly, alphaShape was used to determine the SSD boundaries of channel 2 and then each SSD localization of channel 1 was tested for inclusion inside a channel 2 SSD. The alphaVolume of overlapping localizations from channel1 was then determined for each SSD and the sum of SSD overlap for every SSD in channel 1 value was divided by the total of SSD volumes for channel 1 in that synapse.
Quantal release of glutamate was carried out with MATLAB by using a Monte Carlo algorithm that simulated the stochastic behavior of molecule diffusion and dynamic binding to AMPARs in a complex microenvironment with a time step of 0.5 µs.
SIMULATION OF AMPAR DISTRIBUTION
Considering its unique structure, the CA1 could be considered as a large parallel arrangement of a few hundred active zones aligned to the corresponding postsynaptic sites. Thus, the extracellular space between the presynaptic and postsynaptic membrane was regarded as two paralleled coaxial cylinders of 0.5 µm length, with a 15 nm synaptic cleft between the cylinders.
We adopted a previous model to describe the number of different internal states of GluA1. The radiuses of nano- and synaptic clusters of GluA1 were calculated based on STORM data. A total of 100 GluA1 were placed as assigned with the ratio estimated from STORM (Table 1).
SIMULATION OF GLUTAMATE RELEASE AND AMPAR ACTIVATION
The initial fusion pore conductance of a single vesicle is >375 pS 27 and the relationship between transmitter flux and conductance32 permits a calculation of vesicular expansion time (τ=73 γ^(-1) µs, where γ is the fusion pore conductance in nS) of 0.2 ms to release its all transmitters. The number of glutamate molecules in the vesicle was set to 3000. After release, glutamate molecules do Brownian motion at a diffusion rate of 0.4 µm2 / ms.
When glutamate hits the membrane or even AMPAR, it will not be bound but reflected. A 9-states AMPAR reaction scheme was taken from a previous study. An AMPAR was run against the glutamate transients to calculate the open probability of individual AMPAR. Every AMPAR was regarded as a circular area with a radius of 10 nm and its internal state depending on the number of glutamates hitting this area during a one-time step. The effect of glutamate binding to GluAs is negligible for the much greater number of glutamate molecules (3000) than GluA1 (100). The rate constants of GluA1 were initially set as previous study31 and were adjusted within the constraint of microscopic reversibility.
Transporters were uniformly distributed on the glial sheath which surrounded the synapse. The default density of transporters was 2000 / µm2 and the distance between synaptic edge and glia was 40 nm.
The traces shown here were mean values of 160 runs with release sites randomly distributed through the active zone with a radius equal to nanocluster. All the default parameters we used are listed in Table 1 unless otherwise stated. The 10-90% rise time and decay time were calculated by fitting the rise and decay phases of EPSCs with double exponential functions. EPSC at time t is generated by
I(t)=[g×n(t)]×(V_m-V_GluAs) (1)
where g is the single-channel conductance set at 31 pS for GluA129, n(t) is the number of open GluA1 at time t, Vm is the resting membrane potential and VGluAs is the reversal potential of GluA1.
Code generated for this simulation is available at https://github.com/Han-y/Synapse-Model-for-Aoto-Lab and code generated for STORM analysis are available at https://github.com/Brian3342/Aoto_Lloyd_2023.

For manuscripts utilizing custom algorithms or software that are central to the research but not yet described in published literature, software must be made available to editors and reviewers. We strongly encourage code deposition in a community repository (e.g. GitHub). See the Nature Portfolio guidelines for submitting code & software for further information.

Data

Policy information about availability of data

All manuscripts must include a data availability statement. This statement should provide the following information, where applicable:

- Accession codes, unique identifiers, or web links for publicly available datasets
- A description of any restrictions on data availability
- For clinical datasets or third party data, please ensure that the statement adheres to our policy

Source data generated in this study are provided in the Source Data file. Raw data will be provided upon request.

Human research participants

Policy information about studies involving human research participants and Sex and Gender in Research.

Reporting on sex and gender	N/A
Population characteristics	N/A
Recruitment	N/A
Ethics oversight	N/A

Note that full information on the approval of the study protocol must also be provided in the manuscript.

Field-specific reporting

Please select the one below that is the best fit for your research. If you are not sure, read the appropriate sections before making your selection.

☒ Life sciences ☐ Behavioural & social sciences ☐ Ecological, evolutionary & environmental sciences

For a reference copy of the document with all sections, see nature.com/documents/nr-reporting-summary-flat.pdf

Life sciences study design

All studies must disclose on these points even when the disclosure is negative.

Sample size	For all experiments, at least 3 independent cultures were used of mixed-sex pups. A minimum of 50 synapses were imaged per culture from multiple randomly selected regions from multiple coverslips. A sample size of approximately 100 synapses per condition was utilizing as has been widely used in the past and allows for the detection of 20% changes in synaptic parameters. For each region, 20,000 frames were collected.
Data exclusions	Localizations with a sigma of greater than 40nm in xy or 80nm in the z dimension were excluded from further analysis. Synaptic compartments for GluA1, RIM1, and PSD-95 were excluded from analysis if they contained 0 SSDs and/or less than 50 localizations, as has been used widely in the past. For transsynaptic alignment, SSDs that were incorrectly translated were excluded from further analysis. For outlier analysis, the ROUT test in GraphPad prism was used with Q=1%. These parameters were set prior to the analysis of data.
Replication	All experiments were repeated in 3 independent cultures which showed replication of observed effects.
Randomization	Cultures were infected and stained randomly throughout the plate to minimize any potential covariate of plate position for any synaptic properties.
Blinding	The experimenters were blinded for data collection and analysis until data was collated for statistical analysis.

Reporting for specific materials, systems and methods

We require information from authors about some types of materials, experimental systems and methods used in many studies. Here, indicate whether each material, system or method listed is relevant to your study. If you are not sure if a list item applies to your research, read the appropriate section before selecting a response.

Materials & experimental systems

n/a	Involved in the study
☐	☒ Antibodies
☐	☒ Eukaryotic cell lines
☒	☐ Palaeontology and archaeology
☐	☒ Animals and other organisms
☒	☐ Clinical data
☒	☐ Dual use research of concern

Methods

n/a	Involved in the study
☒	☐ ChIP-seq
☒	☐ Flow cytometry
☒	☐ MRI-based neuroimaging

Antibodies

Antibodies used

Antibodies

Mouse anti-PSD-95 ThermoFisher Cat# MA1-045; RRID:AB_325399; clone 6G6-1C9; lot XE341719
Rabbit anti-RIM1 Synaptic Systems Cat# 140 003; RRID:AB_887774; lot 6-28
Mouse anti-GluA1 Sigma Cat# ZMS1007; RRID: AB_2923292; clone RH95; lot Q35504055
Mouse anti-V5 ThermoFisher Cat# 46-0705; RRID:AB_2556564; clone SV5-Pk1; lot 2611676
Mouse anti-HA AF647 ThermoFisher Cat# 26183-A647; RRID:AB_2610626; clone 2-2.2.14; lot WF329941
Rabbit anti-LRRTM2 AF647 Bioss Cat# bs-11877R; RRID:AB_2923293; lot BA12061755
Rabbit anti-GluD1 AF647 Bioss Cat# bs-12095R; RRID:AB_2923294; lot BA12067820
Guinea pig anti-GluD1 Frontier Institute GluD1C-GP-Af840; RRID:AB_2571759; lot 245
Chicken anti-Homer1 Synaptic Systems Cat# 160 006; RRID:AB_2631222; lot 1-15
Rabbit anti-VGAT Synaptic Systems Cat# 131 002; RRID:AB_887871; lot 2-49
Guinea pig anti-VGluT1 Synaptic Systems Cat# 135 304; RRID:AB_887878; lot 1-56
Rabbit anti-pan Nrxn Frontier Institute Cat# MSFR104630; RRID:AB_2571817; lot 301
Donkey anti-Guinea Pig AF647 Jackson Immunoresearch Cat# 706-605-148; RRID:AB_2340476; lot 145782
Donkey anti-Mouse CF568 Biotium Cat# 20105; RRID:AB_10853136; lot 19C1004
Goat anti-Chicken CF568 Biotium Cat# 20104; RRID:AB_10853460; lot 16C0818
Goat anti-Chicken AF488 Jackson Immunoresearch Cat# 103-545-155; RRID:AB_2337390; lot 165791

Donkey anti-Guinea Pig CF568 Biotium Cat#20377; RRID:AB_2934264; lot 17C0322
Guinea pig anti-RIM1/2 Synaptic Systems Cat# 140 205; RRID:AB_2631216; lot 1-5
Rabbit anti-Homer1 Synaptic Systems Cat# 160 003; RRID:AB_887730; lot 1-32
Mouse anti-GluA2 Biolegend Cat# 810501; RRID:AB_2564751; clone L21-32; lot B219629
Mouse anti-βActin Millipore Sigma Cat# A5441; RRID:AB_476744; clone AC-15; lot 0000126949
Rabbit anti-V5 Cell Signaling Technology Cat# D3H8Q; RRID:AB_2923295; lot 7
Donkey anti-mouse AF790 Jackson Immunoresearch Cat# 715-655-150; RRID:AB_2340870; lot 163480
Donkey anti-rabbit AF680 Jackson Immunoresearch Cat# 711-625-152; RRID:AB_2340627; lot 160238
Donkey anti-guinea pig AF680 Jackson Immunoresearch Cat# 706-625-148; RRID:AB_2340478; lot 162403
Rabbit anti-Human control IgG Jackson Immunoresearch Cat # 309-005-008; RRID:AB_2339626; lot 132102

Validation

Mouse anti-PSD-95 ThermoFisher Cat# MA1-045; RRID:AB_325399 PMID:10488080, PMID:10648730, PMID:10844022, PMID:10844024, PMID:11018053, PMID:11069941, PMID:11896155, PMID:12415013, PMID:12603262, PMID:12695502, PMID:12834111, PMID:1419001, PMID:14642282, PMID:14691139, PMID:14724236, PMID:15071120, PMID:15317862, PMID:15345244, PMID:15355980, PMID:15380068, PMID:15537891, PMID:15574493, PMID:15588724, PMID:15634787, PMID:15707899, PMID:15718246, PMID:15758177, PMID:15800193, PMID:15930126, PMID:15976529, PMID:16041714, PMID:16298350, PMID:16299499, PMID:16386336, PMID:16467530, PMID:16476664, PMID:16483723, PMID:16515559, PMID:16572456, PMID:16706833, PMID:16738247, PMID:16795052, PMID:16802322, PMID:16815335, PMID:16885225, PMID:16930401, PMID:16957582, PMID:17029265, PMID:17045249, PMID:17088214, PMID:17189701, PMID:17251419, PMID:17360912, PMID:17420446, PMID:17507558, PMID:17532795, PMID:17553983, PMID:17626197, PMID:17626212, PMID:17652593, PMID:17920452, PMID:18003830, PMID:18049715, PMID:18215622, PMID:18279318, PMID:18626093, PMID:18780974, PMID:18957222, PMID:18957537, PMID:19029062, PMID:19047646, PMID:19087171, PMID:19141314, PMID:19146814, PMID:19187438, PMID:19190776, PMID:19231096, PMID:19244205, PMID:19285470, PMID:19458219, PMID:19508696, PMID:19691842, PMID:19726651, PMID:19843561, PMID:19951292, PMID:20356829, PMID:20371700, PMID:20392935, PMID:20452978, PMID:20456011, PMID:20519524, PMID:20554866, PMID:20573903, PMID:20648651, PMID:20720104, PMID:20731764, PMID:20831617, PMID:20847396, PMID:20926668, PMID:21068315, PMID:21147983, PMID:21173224, PMID:21209221, PMID:21236347, PMID:21248129, PMID:21262467, PMID:21289286, PMID:21324980, PMID:21331566, PMID:21719075, PMID:21752999, PMID:21935408, PMID:21953547, PMID:22166974, PMID:22191421, PMID:22198376, PMID:22198502, PMID:22279193, PMID:22286174, PMID:22302825, PMID:22347480, PMID:22351628, PMID:22355283, PMID:22369234, PMID:22375696, PMID:22404518, PMID:22411227, PMID:22412961, PMID:22523092, PMID:22542739, PMID:22579289, PMID:22649244, PMID:22668780, PMID:22740692, PMID:22776425, PMID:22844492, PMID:22875941, PMID:22973009, PMID:23015446, PMID:23077044, PMID:23182244, PMID:23276635, PMID:23358245, PMID:23382131, PMID:23479425, PMID:23487039, PMID:23525042, PMID:23567299, PMID:23658157, PMID:23675510, PMID:23793416, PMID:23818861, PMID:23821558, PMID:23827676, PMID:23946402, PMID:24032433, PMID:24046442, PMID:24055117, PMID:24114974, PMID:24155300, PMID:24156266, PMID:24223993, PMID:24269729, PMID:24285894, PMID:24347167, PMID:24394804, PMID:24418105, PMID:24509844, PMID:24553943, PMID:24560713, PMID:24587109, PMID:24588402, PMID:24589888, PMID:24705599, PMID:24753442, PMID:24882000, PMID:24885504, PMID:25014177, PMID:25104558, PMID:25369168, PMID:25529631, PMID:25589756, PMID:25653381, PMID:25773364, PMID:25834051, PMID:26203146, PMID:26391661, PMID:26490870, PMID:26550561, PMID:26571461, PMID:26650351, PMID:26700428, PMID:26843334, PMID:26880549, PMID:26888934, PMID:27026294, PMID:27073215, PMID:27117477, PMID:27121581, PMID:27129212, PMID:27186643, PMID:27187935, PMID:27189977, PMID:27194588, PMID:28231043, PMID:28452394, PMID:28520937, PMID:28736134, PMID:28829741, PMID:28936725, PMID:29024660, PMID:29024665, PMID:29793972, PMID:30020076, PMID:30100184, PMID:30125942, PMID:30209204, PMID:30225353, PMID:30344043, PMID:30355633, PMID:30683685, PMID:30685224, PMID:30826520, PMID:31068362, PMID:31578233, PMID:31628178, PMID:31836739, PMID:32068976, PMID:32801156, PMID:7569905, PMID:9822767, PMID:9870942, PMID:9883737, PMID:9971750
Rabbit anti-RIM1 Synaptic Systems Cat# 140 003; RRID:AB_887774 KO verified; tested species: mouse
Mouse anti-GluA1 Sigma Cat# ZMS1007; RRID: AB_2923292 Each ZooMAb antibody is manufactured using our proprietary recombinant expression system, purified to homogeneity, and precisely dispensed to produce robust and highly reproducible lot-to-lot consistency. Only top-performing clones are released for use by researchers. Each antibody is validated for high specificity and affinity across multiple applications, including its most commonly used application.
Mouse anti-V5 ThermoFisher Cat# 46-0705; RRID:AB_2556564 This antibody is functionally tested against 20 ng of an E. coli expressed fusion protein containing a V5 epitope using a chemiluminescent substrate at a 1 minute exposure. This antibody has also been tested in Western blot against 25 ng of recombinant Positope™ protein. The Positope™ control protein is a 53 kDa recombinant protein that contains seven epitope tags, including His (C-term), HisG, c-myc, and V5. Low background was observed using chemiluminescent or alkaline phosphatase reagents for detection. There are 144 citations for this antibody for ICC.
Mouse anti-HA AF647 ThermoFisher Cat# 26183-A647; RRID:AB_2610626 PMID:12119359, PMID:16818521, PMID:16925789, PMID:19332560, PMID:20920456, PMID:23558175, PMID:23961993, PMID:24358235, PMID:24847881, PMID:26446986, PMID:26649940, PMID:27018236, PMID:27535802, PMID:27698019, PMID:27793988, PMID:27898713, PMID:28087630, PMID:28669802, PMID:28867595, PMID:29395063, PMID:29521627, PMID:30375416, PMID:30392931, PMID:30415698, PMID:30576652, PMID:30581135, PMID:30765194, PMID:31318984, PMID:31875563, PMID:31934861, PMID:31955845
Rabbit anti-LRRTM2 AF647 Bioss Cat# bs-11877R; RRID:AB_2923293 Validated using shRNA knockdown (de Wit et al., 2009) of endogenous LRRTM2 in this manuscript.
Rabbit anti-GluD1 AF647 Bioss Cat# bs-12095R; RRID:AB_2923294 Validated by flow cytometry https://www.biossusa.com/products/bs-12095r
Guinea pig anti-GluD1 Frontier Institute GluD1C-GP-Af840; RRID:AB_2571759. K.O. validated PMID: 24872547.
Chicken anti-Homer1 Synaptic Systems Cat# 160 006; RRID:AB_2631222 Specific for Homer 1. According to Soloviev et al. (2000), aa 1 - 180 are present in isoforms a, b, c and d.
Rabbit anti-VGAT Synaptic Systems Cat# 131 002; RRID:AB_887871 KO verified
Guinea pig anti-VGluT1 Synaptic Systems Cat# 135 304; RRID:AB_887878 K.O. validated PubMed: 34876472
Rabbit anti-pan Nrxn Frontier Institute Cat# MSFR104630; RRID:AB_2571817 Trotter et al., 2019
Donkey anti-Guinea Pig AF647 Jackson Immunoresearch Cat# 706-605-148; RRID:AB_2340476 No antibody was detected against non-immunoglobulin serum proteins. The antibody has been tested by ELISA and/or solid-phase adsorbed to ensure minimal cross-reaction with bovine, chicken, goat, syrian hamster, horse, human, mouse, rabbit, rat and sheep serum proteins, but it may cross-react with immunoglobulins from other species.
Donkey anti-Mouse CF568 Biotium Cat# 20105; RRID:AB_10853136 To minimize cross-reactivity, the antibody has been adsorbed

against bovine, chicken, goat, guinea pig, Syrian hamster, horse, human, rabbit, rat and sheep serum proteins.
Donkey anti-Guinea Pig CF568 Biotium Cat#20377; RRID:AB_2934264 To minimize cross-reactivity, the antibody has been adsorbed against bovine, chicken, goat, Syrian hamster, horse, human, mouse, rabbit, and sheep serum.
Goat anti-Chicken CF568 Biotium Cat# 20104; RRID:AB_10853460 To minimize cross-reactivity, the antibody has been adsorbed against bovine, chicken, goat, guinea pig, Syrian hamster, horse, human, rabbit, rat and sheep serum proteins.
Goat anti-Chicken AF488 Jackson Immunoresearch Cat# 103-545-155; RRID:AB_2337390 No antibody was detected against non-immunoglobulin serum proteins. The antibody has been tested by ELISA and/or solid-phase adsorbed to ensure minimal cross-reaction with bovine, chicken, goat, syrian hamster, horse, human, mouse, rabbit, rat and sheep serum proteins, but it may cross-react with immunoglobulins from other species.
Guinea pig anti-RIM1/2 Synaptic Systems Cat# 140 205; RRID:AB_2631216 RIM 2 including splice variants, cross reacts to RIM 1.
Rabbit anti-Homer1 Synaptic Systems Cat# 160 003; RRID:AB_887730 Specific for Homer 1. Cross-reactivity of the serum to Homer 2 and 3 was removed by pre-adsorption with Homer 2 (aa 1 - 176) and Homer 3 (aa 1 - 177).
Mouse anti-GluA2 Biolegend Cat# 810501; RRID:AB_2564751 Each lot of this antibody is quality control tested by Western blotting. For Western blotting, the suggested use of this reagent is 1.0 - 10 µg per ml. For immunohistochemistry on formalin-fixed paraffin-embedded tissue, a concentration range of 1.0 - 10 µg/ml is suggested. It is recommended that the reagent be titrated for optimal performance for each application.
Mouse anti-βActin Millipore Sigma Cat# A5441; RRID:AB_476744
Rabbit anti-V5 Cell Signaling Technology Cat# D3H8Q; RRID:AB_2923295 V5-Tag (D3H8Q) Rabbit mAb recognizes transfected levels of recombinant protein containing the V5 epitope tag.
Donkey anti-mouse AF790 Jackson Immunoresearch Cat# 715-655-150; RRID:AB_2340870 No antibody was detected against non-immunoglobulin serum proteins. The antibody has been tested by ELISA and/or solid-phase adsorbed to ensure minimal cross-reaction with bovine, chicken, goat, syrian hamster, horse, human, mouse, rabbit, rat and sheep serum proteins, but it may cross-react with immunoglobulins from other species.
Donkey anti-rabbit AF680 Jackson Immunoresearch Cat# 711-625-152; RRID:AB_2340627 No antibody was detected against non-immunoglobulin serum proteins. The antibody has been tested by ELISA and/or solid-phase adsorbed to ensure minimal cross-reaction with bovine, chicken, goat, syrian hamster, horse, human, mouse, rabbit, rat and sheep serum proteins, but it may cross-react with immunoglobulins from other species.
Donkey anti-guinea pig AF680 Jackson Immunoresearch Cat# 706-625-148; RRID:AB_2340478 No antibody was detected against non-immunoglobulin serum proteins. The antibody has been tested by ELISA and/or solid-phase adsorbed to ensure minimal cross-reaction with bovine, chicken, goat, syrian hamster, horse, human, mouse, rabbit, rat and sheep serum proteins, but it may cross-react with immunoglobulins from other species.
Rabbit anti-Human control IgG Jackson Immunoresearch Cat # 309-005-008; RRID:AB_2339626 No antibody was detected against non-immunoglobulin serum proteins. The antibody has been tested by ELISA and/or solid-phase adsorbed to ensure minimal cross-reaction with bovine, chicken, goat, syrian hamster, horse, human, mouse, rabbit, rat and sheep serum proteins, but it may cross-react with immunoglobulins from other species.

Eukaryotic cell lines

Policy information about cell lines and Sex and Gender in Research

Cell line source(s)

Cultures were made from mixed sex P0 mouse pups from the genotypes indicated in the manuscript. Additionally, although not discussed, we genotyped and plated neurons from male and female P0 pups (genotyped using SRY specific primers). We did not observe any differences in nanoscale architecture so in conditions were male and female neurons were analyzed separately, the data were combined.

Authentication

Genotyping of P0 pup tail snips after plating.

Mycoplasma contamination

Confirm that all cell lines tested negative for mycoplasma contamination OR describe the results of the testing for mycoplasma contamination OR declare that the cell lines were not tested for mycoplasma contamination.

Commonly misidentified lines
(See ICLAC register)

Name any commonly misidentified cell lines used in the study and provide a rationale for their use.

Animals and other research organisms

Policy information about studies involving animals; ARRIVE guidelines recommended for reporting animal research, and Sex and Gender in Research

Laboratory animals

Nrxn3fl/fl mouse Gift, Dr. Thomas Südhof, Stanford University JAX Strain #:014157; RRID:IMSR_JAX:014157
V5-Nrxn3 mouse This paper N/A
HA-Nrxn1 mouse Gift, Dr. Thomas Südhof, Stanford University N/A
Animals used for culture were used at P0. All other animals used for this study were P40-50.

Wild animals

No wild animals were used in this study.

Reporting on sex

Sex was considered as a biological variable. pups were genotyped using SRY specific primers. In each experiment, male and female cultures were initially made separately, however, we did not observe sex differences in the nanoscale organization of any of our parameters. We also made mixed sex hippocampal cultures and the resulting values were identical to those of individual sexes. We thus pooled data from both sexes.

Field-collected samples

No field-collected samples were used in this study.

Ethics oversight

All procedures were conducted in accordance with guidelines approved by Administrative Panel on Laboratory Animal Care at

Ethics oversight	University of Colorado, Anschutz School of Medicine, accredited by Association for Assessment and Accreditation of Laboratory Animal Care International (AAALAC) (00235).

Note that full information on the approval of the study protocol must also be provided in the manuscript.
